# Teacher Rated School Ethos and Student Reported Bullying—A Multilevel Study of Upper Secondary Schools in Stockholm, Sweden

**DOI:** 10.3390/ijerph14121565

**Published:** 2017-12-13

**Authors:** Bitte Modin, Sara B. Låftman, Viveca Östberg

**Affiliations:** Centre for Health Equity Studies (CHESS), Stockholm University/Karolinska Institutet, SE-10691 Stockholm, Sweden; sara.brolin.laftman@chess.su.se (S.B.L.); viveca.ostberg@chess.su.se (V.Ö.)

**Keywords:** school ethos, effective schools, student, teacher, bullying, cyberbullying, contextual, Sweden

## Abstract

School ethos refers to the school leadership’s purposive efforts to shape and direct the attitudes, values and behaviors needed in order to promote an active learning environment and to prevent the emergence of undesirable behaviors by creating shared meaning and common goals for the school. The aim of this study was to examine how teacher rated aspects of school ethos are linked with manifestations of bullying among 11th grade students. Five teacher-rated sub-dimensions of school ethos (staff stability, teacher morale, structure-order, student focus, and academic atmosphere) were examined in relation to student-reported perpetration of and exposure to traditional school bullying and cyberbullying. The data material combines student and teacher information from two separate data collections performed in 2016, comprising teachers and students in 58 upper secondary schools in Stockholm. Analyses showed that bullying was associated with all but one of the five sub-dimensions of school ethos, namely structure and order for dealing with bullying behaviors at the school. Results are discussed in light of this counter-intuitive finding. Our findings nevertheless lend support to the idea that the social organization of schools, as reflected in their teacher-rated ethos, can affect individual students’ attitudes in a way that prevents the emergence of bullying behavior among students.

## 1. Introduction

An increasing number of scholars highlight the importance of taking school-contextual factors into account when trying to understand the causes and consequences of school bullying [1,2,3]. One important reason for this upsurge of interest is that anti-bullying programs tend to rely on the presumption that bullying can be combatted by modifying features of the school environment [4]. According to a recent systematic review on this topic, typical school features that have been investigated as predictors of bullying are organizational conditions (e.g., school size and location), sociodemographic characteristics, anti-bullying norms, teacher support, class management, degree of academic pressure, and school climate, all of which demonstrate consistent links to peer bullying with the only exception being certain organizational characteristics [3].

A frequently used definition of school bullying was initially phrased by Dan Olweus [5] (p. 9: “A student is being bullied or victimized when he or she is exposed, repeatedly and over time, to negative actions on the part of one or more students”. Such negative actions can take different forms and may be physical or verbal, direct or indirect, but they all serve the purpose of causing harm or discomfort to the targeted individual [6]. In the school setting these actions are generally expressed face-to-face, for example through explicit disapproval, teasing, ostracism, or physical harm. An additionally important element of bullying is the actual or perceived imbalance of power between the involved parties, rendering it more difficult for the victim to defend him- or herself [7]. Cyberbullying refers to a specific form of bullying that can be expressed as “the use of electronic communication technologies to bully others” [8]. While there is a considerable overlap in the attributes of cyberbullying and traditional bullying, the former distinguishes itself with some unique features. Firstly, acts of cyberbullying may occur anywhere and anytime. Secondly, the audience is undefined and potentially very large, and thirdly, the perpetrators have greater possibility of remaining anonymous compared to perpetrators of traditional bullying [9]. Regarding the element of repetition as an important component of (traditional) bullying, a single act of cyberbullying can potentially be repeated innumerably through e.g., the digital sharing of images. Exposure to bullying is closely related to internalizing health problems [10,11], whereas bullying perpetration tends to be more strongly associated with externalizing problems [12,13].

A frequently investigated contextual predictor of peer bullying is school climate. In a meta-analysis of individual and contextual determinants of bullying and victimization [14], school climate in terms of student-reported sense of belonging to the school and degree of respect and fair treatment from school staff, was identified as an important predictor of both bullying victimization and perpetration. In addition, school climate in terms of student-reported connectedness to their school and perception of the climate as being trusting, fair, and pleasant [8], as well as commitment to school, feeling safe at school, and sense of belonging to school [15], emerged as robust and significant predictors of both perpetration of and victimization from cyberbullying in two separately performed meta-analyses. To capture its “contextual effects” on outcomes such as bullying, students’ perceptions of the school climate are commonly aggregated to the class- or school-level [16,17]. For instance, a large-scale study, based on ninth-grade students in Stockholm municipality, found that classes where a high proportion of students claimed to be aware of the school rules, and classes with high shares of students stating that adults intervene against bullying, also had smaller shares of students who reported having been bullied during the past school year [17].

Although students constitute a valuable source of information when trying to identify school-contextual features of importance for peer bullying, a major advantage of using indicators retrieved from other sources than the students themselves is that it considerably reduces the bias related to common method variance [18]. Such contextual measures can, for example, consist of administrative data or teacher reports of conditions at the school. By using information retrieved from the National Student Database and the school inspectorate agency in the UK (Ofstead), as well as teacher reported information on various school policies, Mujis [19] found that schools that kept records of bullying incidents both inside and outside the confines of the classroom also displayed less school bullying—as did schools with a high quality leadership and management in general. Levels of bullying perpetration and victimization were also lower in schools characterized by equality of opportunity and social cohesion, something which Mujis interprets as a possible reflection of the school’s ethos [19]. Bevilacqua and colleagues also used Ofstead reports to assess the importance of school leadership for the occurrence of bullying among seventh grade students in 40 English schools [20]. Results showed that students attending schools that had been rated as “Outstanding” reported less victimization from both cyberbullying and traditional bullying compared to students in schools rated as “Good”. Teachers’ ratings of the school’s professional culture have also been found to predict bullying rates in Swedish [21] and Norwegian student populations [22,23].

While a large body of research has investigated the role of school climate for peer bullying, considerably fewer studies of the corresponding kind have been conducted based on the related concept of school ethos. This is what the present study aims to do. School ethos is a contextual concept that broadly refers to the beliefs, values, and norms permeating the school and manifesting themselves in the way that teachers and students relate, interact, and behave towards each other [24,25,26]. The concept is part of a more comprehensive theory of school effectiveness, claiming that certain schools are more successful than others in creating the shared meaning and common goals needed in order to introduce adolescents into adulthood and to counteract negative effects of external factors. Important features of effective schools are a pedagogy that focuses on clear goals, constructive feedback to the students, providing a safe and orderly environment, a strong academic focus, and positive home-school relations [27,28,29,30]. Empirical studies have shown that schools with such features are characterized by higher school performance [24,25], lower levels of delinquency [31] and less alcohol and drug use among their students [32].

Ideally, a school’s ethos should also help to shape and direct student behavior in a way that prevents the emergence of bullying. As pointed out by Bradshaw and Johnson [1], the common norms, beliefs and attitudes within a school can provide a more or less favorable environment for “cultures of bullying” to develop [1], and a school’s bullying record can therefore be assumed to reflect its overall functioning. The teachers’ attitudes towards bullying are of course central to combatting these types of behaviors [33], but perhaps even more important is the way in which they, collectively set the standards for behavior and day-to-day interaction at the school through their own practice [34]. Of the few existing studies that exist in this area of research, a cross-sectional investigation of English seventh grade students found that “faith schools” had a significantly lower prevalence of bullying compared to mainstream state schools [20]. According to the authors, this supports the notion that elements of the school’s ethos are protective against bullying. Another study looked at Steiner (or Waldorf) schools in the South of England as an example of a system with a strong and non-competitive ethos, and found that the level of bullying was much lower than that of the national norm despite the fact that many students came to the school because they had been victimized elsewhere [35]. However, a recently conducted evaluation of an intervention aimed at changing the ethos in a rural Canadian high school by changing teaching practices, orientation processes as well as recognition and reward mechanisms, did not manage to provide any support for a reduction in school bullying two years after the intervention took place [36].

In this study, we proceed from the idea that the concept of school ethos is distinct from that of school climate in that it reflects a contextual property emerging from a higher level in the school structure than school climate does. In the literature, school climate generally refers to the social interaction among students and between students and teachers [37], with most empirical studies proceeding from student-rated information when operationalizing the concept [14,15,16,17]. This mode of procedure takes account of the students’ subjective experience, and can thus be seen as capturing a contextual feature perceived at the student-level. The concept of effective schools places a much greater focus on the importance of purposeful leadership. It points to a property that is imposed from above—from a higher level in the school structure. In order to capture a school’s ethos properly, therefore, one needs to obtain information from other sources than the students themselves. Here one needs to ask representatives of the school who are better equipped to answer these types of questions, for example teachers or principals. To this end, we conducted a survey among all of Stockholm’s upper secondary school teachers in the spring of 2016, which we have linked to existing student information on bullying from the same year. The aim is to explore how teacher-rated indicators of school ethos correspond with various expressions of bullying among individual students. Five sub-dimensions of school ethos were examined in relation to student perpetration of and exposure to traditional bullying and cyberbullying: staff stability; teacher morale; structure and order for dealing with unwanted behavior; student focus; and academic atmosphere.

## 2. Materials and Methods

### 2.1. Data

**Figure 1:**
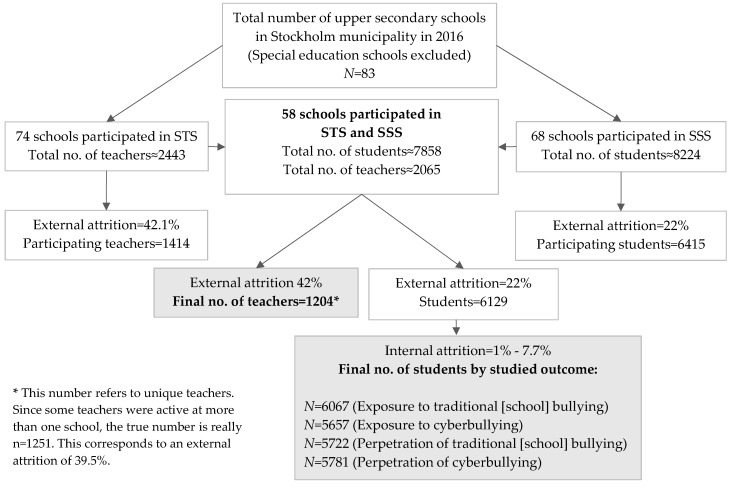
Overview of the process by which the final numbers of study subjects were selected. * Descriptive statistics are based on the original measures, i.e., before they were z-transformed.

The data material combines student and teacher information from two separate data collections performed in 2016, comprising 58 upper secondary schools. Student information comes from the Stockholm School Survey (SSS), a total sample of students in the second grade of upper secondary school (17–18 years) in all public and most independent schools in Stockholm municipality (*N* = 6129; response rate = 88%). The Stockholm Teacher Survey (STS) was carried out by our research group through a web-based questionnaire sent to all upper secondary-level teachers (i.e., first-, second-, and third-grade teachers) in the participating schools (*N* = 1204; response rate 58%). The final numbers of study subjects varies between *N* = 6067 and *N* = 5657 depending on the type of bullying outcome that is analyzed. An overview of the process by which the final four study samples were selected is presented in Figure 1.

### 2.2. Measures

#### 2.2.1. Dependent Variables

Descriptive statistics for all of the variables used in the analyses are presented in Table 1. Four manifestations of peer bullying constitute the outcome variables. The first identifies students’ exposure to traditional school bullying at least twice a month during the past school year through the question “*How often have you been bullied or harassed at school this year?*” with response categories “*I haven’t been bullied*”, “*It’s happened occasionally*”, “*2 or 3 times a month*”, “*About once a week*”, and “*Several times a week*”. Only 2.3% of the students fell into this category. The second measure takes account of exposure to cyberbullying during the past school year through the question “*Have you been bullied or harassed on the Internet or by text messaging (SMS/MMS) this school year?*” Here, the question is constructed in such a way that it is not possible to take account of the number of times this has occurred since the response categories are either “*Yes*”, “*No*” or “*Don’t know*”. This also leads to a higher share of students, 7.3%, reporting having experienced this.

The third indicator concerns perpetration of traditional school bulling on at least an occasional basis during the past school year by asking “*Have you taken part in bullying or harassment of other students during the past school year?*” with the following response categories: “*No*”, “*Yes*, *occasionally*”, “*Yes*, *2 or 3 times a month*”, “*Yes*, *about once a week*”, “*Yes*, *several times a week*”, and “*Don’t know*”. Altogether, 5.0% of the students belong to this category. Finally, we also identified students who reported having been involved in perpetration of cyberbullying during the past school year through the question “*Have you taken part in the bullying or harassment of other students on the Internet or by text messaging this school year?*”. Here too, the response categories are either “*Yes*”, “*No*” and “*Don’t know*”, with 3.0% admitting to having done so during the past year.

#### 2.2.2. Independent Variables

Five teacher-rated sub-dimensions of the school’s ethos constitute the main predictor variables in this study. The first was rated on a 4-graded scale ranging between “*Agree completely*” to “*Disagree completely*”, and the remaining four according to a 5-graded scale ranging between “*Strongly agree*” to “*Strongly disagree*”.

The first sub-dimension concerns the school’s degree of staff stability in terms of the level of sick-leave among teachers, staff turnover and the frequency of substitute teachers at the school. The second takes account of teacher morale in terms of whether the teachers’ have a strong work ethic, work with great enthusiasm, take pride in their school and feel confident as classroom leaders. The third concerns structure and order for dealing with unwanted behavior through questions about the schools’ value system, whether the school actively works on issues such as violence, bullying and harassment among students, whether teachers feel confident about what they may and may not do if violent situations arise among students, and whether the rules for order and conduct are clear at the school. A fourth aspect takes account of the teachers’ degree of student focus in terms of positive feedback to, and high expectations of the students, as well as whether the teachers take their time with students even if they want to discuss something other than schoolwork, and also whether the students are treated with respect. The fifth sub-dimension, finally, tries to capture the school’s academic atmosphere by asking the teachers to assess whether the school provides a stimulating learning environment and whether the students’ motivation is a stimulating part of work.

The teachers’ ratings of the schools’ overall ethos and its’ five sub-dimensions were aggregated to the school-level mean, and then z-transformed to have a mean of 0 and a standard deviation of 1. Cronbach’s alpha for the five sub-dimensions were 0.55 (staff stability, 3 items), 0.93 (teacher morale, 4 items), 0.92 (structure and order, 4 items), 0.79 (student focus, 4 items) and 0.89 (academic atmosphere, 2 items). The overall measure of school ethos (17 items) had a Cronbach’s alpha of 0.94.

#### 2.2.3. Control Variables

Some basic sociodemographic background characteristics of the students were included in the statistical analyses as control variables. Gender was assessed by the question “*Are you a boy or a girl?*” Students who refrained from answering the question (3.4%) were kept as a separate category since they were much more likely to report being victims of bullying. Parental education was measured by the question “*What is the highest education your parents have?*” with separate response categories referring to mothers’ and fathers’ education. Four categories were singled out: students with two, one, or no university-educated parent(s) as well as students who refrained from answering the question. Migration background was captured by asking “*How long have you lived in Sweden?*” The response categories were: “*All my life*”, “*10 years or more*”, “*5–9 years*” and “*Less than 5 years*”. The two latter categories were merged due to small numbers.

### 2.3. Ethics

In the Stockholm School Survey, students provide no information on personal identification and hence the data are filled in anonymously. As a consequence, the SSS data are not subject to consideration for ethical approval according to a decision by the Regional Ethical Review Board of Stockholm (2010/241-31/5). The Review Board has provided ethical permission for the Stockholm Teacher Survey (2015/1827-31/5).

### 2.4. Statistical Method

The data material consists of information collected at two different levels of the school structure. The teacher-rated measures take account of conditions at the school-level, whereas the background characteristics and outcome variables refer to individual students’ circumstances. Therefore, multilevel modelling in terms of two-level binary logistic regression analysis was applied, using the *xtmelogit* command in Stata 14. By estimating an “empty model” it is possible to evaluate if there is variation in bullying across schools. Such a model does not include any independent variables, but assesses the variation in the outcome separately for each level (student; school). From this information, the Intra Class Correlation (ICC) can be calculated. ICC for binary outcomes gives approximate information about the percentage of the total variation in the outcome variable that can be attributed to the school-level.

## 3. Results

**Figure 2:**
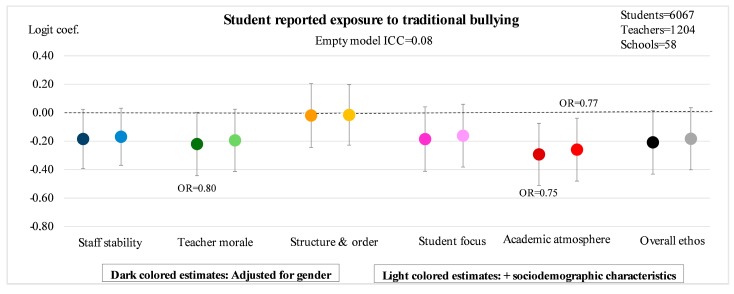
Student reported exposure to traditional bullying according to five sub-dimensions of teacher-rated school ethos as well as overall ethos. Estimated logit coefficients from two-level random intercept logistic regression models with 95% confidence intervals.

**Figure 3:**
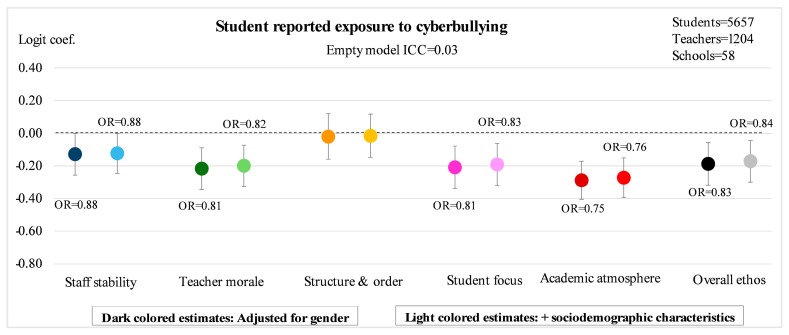
Student reported exposure to cyberbullying according to five sub-dimensions of teacher-rated school ethos as well as overall ethos. Estimated logit coefficients from two-level random intercept logistic regression models with 95% confidence intervals.

**Figure 4:**
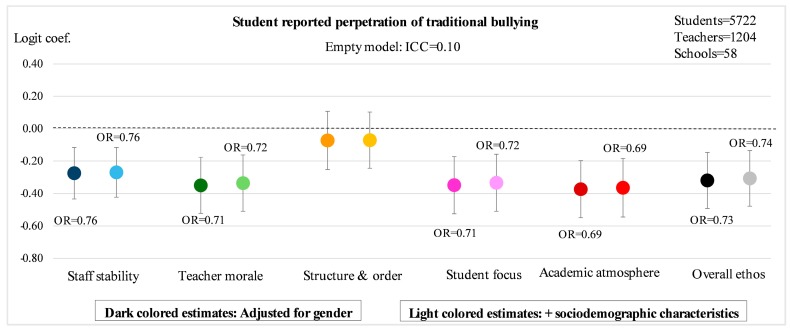
Student reported perpetration of traditional bullying according to five sub-dimensions of teacher-rated school ethos as well as overall ethos. Estimated logit coefficients from two-level random intercept logistic regression models with 95% confidence intervals.

**Figure 5:**
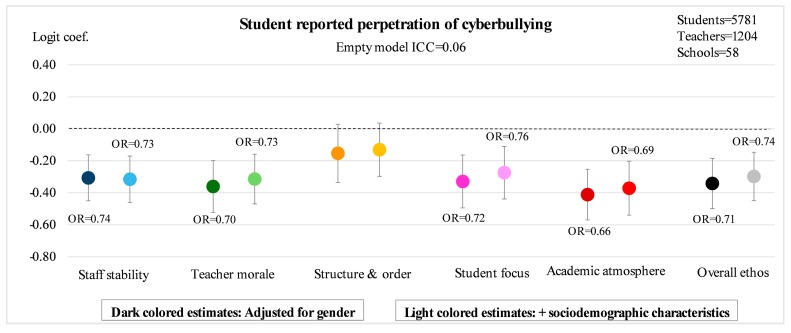
Student reported perpetration of cyberbullying according to five sub-dimensions of teacher-rated school ethos as well as overall ethos. Estimated logit coefficients from two-level random intercept logistic regression models with 95% confidence intervals.

Figure 2, Figure 3, Figure 4 and Figure 5 illustrate the main results of the study. The ICC estimates from the empty models indicate that 8% of the variation in exposure to traditional bullying, and 3% of the variation in exposure to cyberbullying, can be attributed to conditions at the school-level. The corresponding estimates for perpetration of traditional bullying and of cyberbullying are 10% and 6%, respectively. The point estimates in the figures represent logit estimates with 95% confidence intervals. For the statistically significant estimates, the corresponding odds ratios are also reported. Point estimates from two different models are presented for each of the five sub-dimensions as well as for the overall measure of school ethos: the first is adjusted for gender, and the second for gender and sociodemographic background in terms of migration background and parental education.

Figure 2 shows how the five teacher-rated sub-dimensions as well as the overall measure of ethos are associated with individual students’ odds of being exposed to traditional bullying before and after adjustment for sociodemographic background. Here, one should be observant on whether the confidence intervals, illustrated by the vertical lines attached to each of the logit estimates, exceed the value of 0, which is represented by a dotted horizontal line in the figure. This is the case for most of the estimates, which means that they are not statistically significant at the 5%-level. However, the aspects representing teacher morale and academic atmosphere are significantly associated with student-reported exposure to traditional bullying in the gender-adjusted model. When the students’ sociodemographic background is adjusted for, however, only the estimate for academic atmosphere remains with a significant effect. The estimate indicates that each standard deviation increase in academic atmosphere is associated with a decreased odds of exposure to traditional bullying corresponding to OR = 0.77 (*p* = 0.021). With the exception of the sub-dimension meant to capture *structure and order*, the remaining aspects of school ethos still indicate a decreased likelihood of exposure to bullying as well, although none of these estimates reach statistical significance.

Figure 3 reveals that all sub-dimensions of school ethos, except the one representing *structure and order*, are significantly associated with students’ odds of being exposed to cyberbullying. The estimates are very similar to those of victimization from traditional bullying, but with narrower confidence intervals. Here too, the school’s academic atmosphere emerges as the sub-dimension most strongly associated with victimization (OR = 0.76, *p* < 0.001), but staff stability (OR = 0.88, *p* = 0.049), teacher morale (OR = 0.82, *p* = 0.002) and student focus (OR = 0.83, *p* = 0.003) demonstrate substantial effect sizes in the fully adjusted models as well. The overall measure of school ethos points to a decreased odds of exposure to cyberbullying corresponding to OR = 0.84 (*p* = 0.003) for each standard deviation’s increase in school ethos. Calculated the other way around, a standard deviation decrease in overall school ethos is associated with a close to 19% increased odds of cyberbullying victimization among the students at the school.

Perpetration of traditional bullying is even more strongly linked to teacher-rated aspects of school ethos (Figure 4). This is true for all of the four sub-dimensions of *staff stability*, *teacher morale*, *student focus* and *academic atmosphere*, which demonstrate odds ratios in the range of 0.69–0.76 in the fully adjusted models.

Calculated the other way around, this corresponds to an increased odds of 31–45% of being a perpetrator of bullying for each standard deviation decrease in in any of these four indicators of school ethos. However, no significant effect of structure and order is seen here either. Taken together, one standard deviation increase in teachers’ overall ratings of the school ethos is associated with a decreased occurrence of self-reported bullying perpetration corresponding OR = 0.74 (*p* = 0.032).

Regarding self-reported perpetration of cyberbullying, finally, the picture looks pretty much the same as that for traditional bullying perpetration (Figure 5). For all sub-dimensions of school ethos, except the one for structure and order, a significantly lower prevalence of bullying behavior can be noted as school ethos increases. Academic atmosphere stands out as a slightly stronger predictor of perpetration of cyberbullying (OR = 0.69, *p* < 0.001) compared to staff stability (OR = 0.73, *p* < 0.001), teacher morale (OR = 0.73, *p* < 0.001) and student focus (OR = 0.76, *p* < 0.001) in the fully adjusted models. Calculated the other way around, a standard deviation decrease in overall school ethos is associated with a 35% increased odds of being a perpetrator of bullying (OR = 0.74, *p* < 0.001).

## 4. Discussion

This study investigated how five teacher-rated aspects of school ethos corresponded with students’ experiences of bullying in 58 upper secondary schools in Stockholm, while taking individual sociodemographic covariates into account. Results showed that victimization from cyberbullying decreased significantly with increasing levels of schools’ staff stability, teacher morale, student focus and academic atmosphere. Although this pattern of associations were almost identical for victimization from traditional bullying, the confidence intervals were wider, which left only academic atmosphere as a significant predictor in the fully adjusted model. Teacher-rated aspects of school ethos were even more strongly related to students’ reports of bullying perpetration, once again with staff stability, teacher morale, student focus and academic atmosphere as significant predictors of both traditional bullying and cyberbullying perpetration. These findings corroborate with previous studies showing that teacher-rated aspects of school policies and practices [19] as well as of the school’s professional culture in terms of leadership, teacher affiliation and collaborative activities [22,23], are related to students’ reports of victimization from, and perpetration of, bullying at the school. They are also in agreement with earlier studies that have linked secondary data on school leadership quality to student-reported experiences of bullying [19,20] and cyberbullying [20], demonstrating lower bullying rates in schools rated as having a high quality by the school inspectorate agency in UK (Ofstead reports).

In one respect, however, our findings are in contrast to previous research in the field [19,38]. Thus, while we found support for a link to peer bullying from four of the five investigated sub-dimensions of ethos, there was a consistent lack of any corresponding effect from the aspect of *structure and order*. This ‘non-result’ is perhaps the most intriguing finding of this study given the items that this sub-dimension takes into account, namely (1) At this school we have a value system which is clear to students; (2) At this school we actively work on issues such as violence, bullying and harassment among students; (3) At this school the teachers feel confident about what they may and may not do if violent situations arise among students; and (4) The rules for order and conduct are clear at this school. The complete lack of effect from this measure on peer bullying appears counter-intuitive, as this literally is what it is meant to do. In the study by Mujis [19], teacher assessed school policies and practices directly targeting or dealing with bullying were the predictors with the strongest explanatory power for bullying. The conclusion that school-based anti-bullying programs are indeed effective was also drawn by the authors of an extensive systematic and meta-analytic review of 53 different program evaluations [38] as a response to two previously conducted reviews reporting little effects of such programs [39,40].

A possible explanation to our findings could be related to the fact that Swedish schools, since 2006, are legally obliged to have an anti-bullying policy [41]. To meet these new demands of accountability, many schools have implemented anti-bullying programs available on the open market [42], resulting in a plethora of different bullying prevention programs being applied in Swedish schools today. The different components of these programs are, however, not equally successful [42,43]. However, regardless of their effectiveness, teachers are still likely to (correctly) report that their school actively works on detecting, preventing and intervening against bullying when referring to the anti-bullying program and declared values of their school’s official documents. What our results indicate, however, is that a school’s value statement to actively work against bullying behavior may not be enough for it to actually come into force and make a difference at the school. This notion corresponds well with Graham’s [30] observation that there often seem to be a ‘gap’ between the rhetoric of the school’s articulated ethos and the actual experiences of teachers and students in studies carried out on this topic. Glover and Coleman [44], in their discussion of how improvement and change of ethos can be achieved, argue that schools with a clear set of values—monitored to ensure that they are integrated in policy and practice—are the ones bound to be the most successful.

Sweden has a history of very low bullying rates compared to other European and North American countries [45,46,47], although rates vary considerably between schools. In this study of upper secondary school students (ages 17–18 years), 2.3% were identified as victims, and 5.0% as perpetrators of traditional bullying. Notably, a more conservative cut-off was used for victims (at least 2–3 times a month) than for perpetrators (occasionally). Cyberbullying victimization and perpetration was reported by 7.3% and 3.0% of the students, respectively. Our results indicated that 8% of the unexplained variance in victimization of traditional bullying could be attributed to the school-level, while the corresponding figure for traditional bullying perpetration was 10%. For understandable reasons, the between-school variation in cyberbullying was smaller, corresponding to 3% for victimization and 6% for perpetration. Although cyberbullying is not confined to the school setting, schools are increasingly required to address this form of bullying. As recognized by Williford and Depaolis [48], bullying that starts online may continue the next day in school as the students discuss the incident with each other [48].

The school is an important social arena in young people’s lives where patterns of social relations are constantly negotiated and refined. Aspects of status and power are important components in the establishment of peer relations, and bullying can sometimes be an effective strategy to climb the status ladder. Status goals have in fact been put forward as an important driving force behind bullying behavior in previous research, especially during adolescence [49]. To foster a school ethos where the competition for status becomes less of a concern among the students is therefore an important step in a schools’ preventive work against bullying. The degree to which potential triggers of bullying, such as competition for status, are “allowed” to manifest themselves in the school setting depends on the school’s collective capacity to constrain such behaviors through prevailing norms. However, to modify school collective norms towards a stronger pressure of conforming to anti-bullying attitudes is a task that requires a conscious and purposeful strategy emanating from a higher level in the school structure than the one made up of individual students.

Interestingly, the four aspects of school ethos that emerged as most important for expressions of peer bullying in this study were largely oriented towards creating good conditions for students’ academic motivation and success. These findings are in line with a recently published longitudinal study on the causal relationship between school climate, school violence and student performance [50]. Rather than pointing towards improved academic outcomes as a function of student-rated school climate and violence, their result suggested a causal link in the opposite direction. This indicates that schools that place a strong effort into improving the academic motivation and success among their students also will decrease violence and improve climate through subsequent improvements in students’ academic performance. Thus, by carrying out their main mission as teachers in a commendable way, teachers may actually help counteract the emergence of bullying behaviors at the school, something which has also been found to be true in relation to crime prevention [51].

A major advantage with the current study is that our measure of school ethos were rated by teachers whereas manifestations of peer bullying were reported by students in the corresponding schools, thereby decreasing the risk of bias due to common method variance. The findings and interpretations of this study should nevertheless be considered with some study limitations in mind. Firstly, the data material is cross-sectional which means that we cannot address questions of causality regarding any of the observed associations. Secondly, there may be confounding due to other school-contextual conditions that we were unable to take into consideration, for example school-specific anti-bullying programs. Thirdly, due to social desirability the two measures of bullying perpetration may have lower validity compared to those reflecting victimization. The association between school ethos and perpetration of traditional as well as cyberbullying were, however, stronger than those for victimization. Fourthly, our findings are based on regional data, which means that they cannot be generalized to 11th grade students in Sweden as a whole, or to other national context. Finally, while the main focus of the present study was on the resulting effect of school ethos, future studies should further elaborate on the qualitative components of the concept as well as on the more precise reasons as to why they tend to differ across schools.

## 5. Conclusions

The findings of this study are in agreement with the underlying assumptions of the effective school literature, namely that efforts at higher levels of the school structure are crucial for providing the necessary conditions for processes at lower levels to come into force. Our results lend support to the idea that the social organization of schools, as reflected in their teacher-rated ethos, can affect individual students’ attitudes in a way that counteracts bullying behaviors at the school. Improving school ethos in terms of academic atmosphere, teacher morale, student focus and staff stability should be considered as a viable complement to school-based interventions to address bullying.

## Figures and Tables

**Table 1:** Descriptive statistics of the variables used in the analyses (*n* = 6067).

Student-Level	*n*	%
Dependent variables		
Exposure to traditional bullying		
No	5928	97.7
Yes	139	2.3
Exposure to cyberbullying ^1^		
No	5246	92.7
Yes	411	7.3
Perpetration of traditional bullying ^2^		
No	5434	95.0
Yes	288	5.0
Perpetration of cyberbullying ^3^		
No	5607	97.0
Yes	174	3.0
Independent variables		
Gender		
Boys	2798	46.1
Girls	3064	50.5
Not reported	205	3.4
Migration background		
Lived in Sweden all life	4937	81.4
Lived in Sweden ≥10 years	530	8.7
Lived in Sweden <10 years	600	9.9
Parental education		
No parent has university education	1228	20.2
One parent has university education	1527	25.2
Both parents have university education	2472	40.7
Not reported	840	13.9
School-level (teacher rated) *	Range	Mean (sd)
Overall school ethos	46.4–77.5	63.0 (6.4)
Sub-dimensions of ethos		
Staff stability	4.7–11.7	8.8 (1.3)
Teacher morale	12.0–19.5	15.9 (1.8)
Structure and order	9.3–19.5	14.5 (1.7)
Student focus	13.9–19.2	16.6 (1.2)
Academic atmosphere	4.6–9.5	7.3 (1.4)

* Descriptive statistics are based on the original measures, i.e., before they were z-transformed. ^1, 2, 3^ Don’t know: *n*^1^ = 410; *n*^2^ = 345; *n*^3^ = 286.
